# Effects of community ambulation training with 3D-printed ankle–foot orthosis on gait and functional improvements: a case series of three stroke survivors

**DOI:** 10.3389/fneur.2023.1138807

**Published:** 2023-05-31

**Authors:** Ji-Eun Cho, Kyeong-Jun Seo, Sunghe Ha, Hogene Kim

**Affiliations:** ^1^Department of Rehabilitation and Assistive Technology, National Rehabilitation Center, Seoul, Republic of Korea; ^2^Department of Physical Education, College of Sciences in Education, Yonsei University, Seoul, Republic of Korea; ^3^Department of Clinical Rehabilitation Research, National Rehabilitation Center, Seoul, Republic of Korea

**Keywords:** stroke, gait, stair, 3D printing, ankle-foot-orthosis (AFO), community

## Abstract

**Introduction:**

Many of the patients using ankle–foot orthoses (AFOs) experience poor fit, pain, discomfort, dislike of the aesthetics of the device, and excessive range of motion restrictions, which diminish the use of AFOs. Although 3D-printed ankle–foot orthoses (3D-AFOs) affect patient satisfaction and overall gait functions such as ankle moment, joint range of motion (ROM), and temporal-spatial parameters, the material properties and manufacturing process of 3D-AFOs are still diverse; the clinical effects of community ambulation using 3D-AFOs and satisfaction in patients with stroke are poorly understood.

**Case description:**

Case 1: A 30-year-old man, with a history of right basal ganglia hemorrhage, presented with marked foot drop and genu recurvatum. Case 2: A 58-year-old man, with a history of multifocal scattered infarction, presented with an asymmetrical gait pattern due to abnormal pelvic movement. Case 3: A 47-year-old man, with a history of right putamen hemorrhage, presented with recent poor balance and a prominent asymmetrical gait pattern due to increased ankle spasticity and tremor. All patients could walk independently with AFOs.

**Interventions and outcomes:**

Gait was assessed under three walking (even, uneven, and stair ascent/descent) and four AFO (no shoes, only shoes, shoes with AFOs, and shoes with 3D-AFOs) conditions. After 4 weeks of community ambulation training with 3D-AFO or AFO, the patients were followed up. Spatiotemporal parameters; joint kinematics; muscle efficiency; clinical evaluations including impairments, limitations, and participation; and patient satisfaction with wearing 3D-AFO were evaluated.

**Results and conclusion:**

3D-AFOs were suitable for community ambulation of patients with chronic stroke and effective on step length, stride width, symmetry, ankle range of motion, and muscle efficiency during even surface walking and stair ascent in patients with chronic stroke. The 4-week community ambulation training with 3D-AFOs did not promote patient participation; however, it increased ankle muscle strength, balance, gait symmetry, and gait endurance and reduced depression among patients with a history of stroke. The participants were satisfied with 3D-AFO's thinness, lightweight, comfortable feeling with wearing shoes, and gait adjustability.

## 1. Introduction

Mobility is limited in most stroke survivors, and the restoration of gait ability is a major task in their rehabilitation (1). Walking difficulties in patients with stroke can be managed using ankle–foot orthoses (AFOs), which stabilize the foot and ankle. Customized AFOs are often prescribed to prevent foot drop causing serious falls, alleviate chronic pain associated with joint deformity, and control the ground reaction force during the stance phase of gait to reduce fatigue (2). However, many AFO users experience poor fit, pain, discomfort, and dislike of the aesthetics of the device; moreover, the design options are limited (3). Although many patients with stroke have an insufficient level of physical function that may cause increased fear of community ambulation, which further leads to depression, they often intentionally avoid AFOs (4).

3D-printed ankle–foot orthoses (3D-AFOs) can be personalized based on individual biomechanical requirements to provide improved function, better fit, and enhanced aesthetics (2). Some recent studies reported that 3D-AFOs affect patient satisfaction and overall gait functions such as ankle moment, joint range of motion (ROM), and temporal-spatial parameters (5–7). The size, thickness, weight, durability, easy usability, walking efficiency, and adjustability should be considered while fabricating 3D-AFOs (2). However, the material properties and physical features of 3D-AFOs are still diverse, and studies on their clinical effects and patient satisfaction are limited (2). Furthermore, only a few studies reported long-term effects of wearing 3D-AFOs (7, 8), and no studies compared the effects of community ambulation with conventional AFOs in patients with stroke.

Community ambulation is an important skill for stroke survivors that incorporates both mobility and social aspects (9). Approximately one-third of stroke survivors with ankle–foot impairment were unable to walk outdoors independently (9, 10). Patients with ankle–foot impairment commonly have limited ability to walk confidently in public venues including uneven terrains, stairways, and slopes, which is closely linked to their participation in community ambulation. Nevertheless, no studies reported whether the use of AFOs in a community environment increases social participation and gait function of patients with stroke.

Herein, we report the effects of community ambulation with 3D-AFOs on gait kinematics, muscle efficiency, and social participation of three patients with chronic stroke after 4-week training.

## 2. Methods

### 2.1. Case presentation

Case 1: A 30-year-old male university student had a history of right basal ganglia hemorrhage with 10 months 12 days duration at the first day of participation. He could walk with AFO on level ground under supervision or stand-by help from one person. He was very motivated to go back to school and meet his friends. The patient had good balance (47 on the Berg balance scale, BBS), slight spasticity diagnosed on the basis of resistance to passive stretch of ankle plantar flexor at rest (1/5 on the modified Ashworth scale, MAS) (11), and proprioceptive dysfunction (Supplementary Table 1). During observational gait analysis, the patient presented with marked foot drop during the swing phase of walking and plantarflexion during the stance phase with appreciable genu recurvatum.

Case 2: A 58-year-old male high school teacher had a history of multifocal scattered infarction 24.5 months ago. He used the prescribed AFO only during level walking and stair ambulation. He was planning to return to work 3 months later. The patient had poor muscle strength (1/5 of the ankle dorsiflexor based on manual muscle test, MMT) (12) and mild spasticity (1/5 on MAS) without any observable proprioceptive dysfunction. He had good balance (45 on BBS) and hemiplegic asymmetrical gait pattern due to abnormal pelvic movement and stiff ankle during walking.

Case 3: A 47-year-old male white-collar worker had a history of right putamen hemorrhage 21.2 months ago. He always had to use the prescribed AFO and cane due to deteriorated ankle spasticity during walking. He was planning to return to the countryside house after discharge. The patient had poor muscle strength (2/5 on MMT), marked spasticity (1+/5 on MAS), and observable proprioceptive dysfunction. He presented with poor balance (43 on BBS) and a prominent asymmetrical gait pattern (weight bearing was biased toward the non-paretic side) due to increased ankle spasticity and tremor. He could walk independently with both AFO and cane at his own slow speed.

This study was approved by the Institutional Review Board of the National Rehabilitation Center, Seoul, South Korea (IRB No. NRC-2021-01-002) and registered for clinical research (No. KCT0007195) prior to the study. All participants provided written informed consent before study enrollment. This study's design was a case series, retrospective study based on single-center trials. All participants were assessed on gait function in four AFO conditions and functional ability and reassessed on functional ability at the end of the 4-week intervention period.

### 2.2. Procedure for manufacturing 3D-printed ankle–foot orthosis

**Figure 1:**
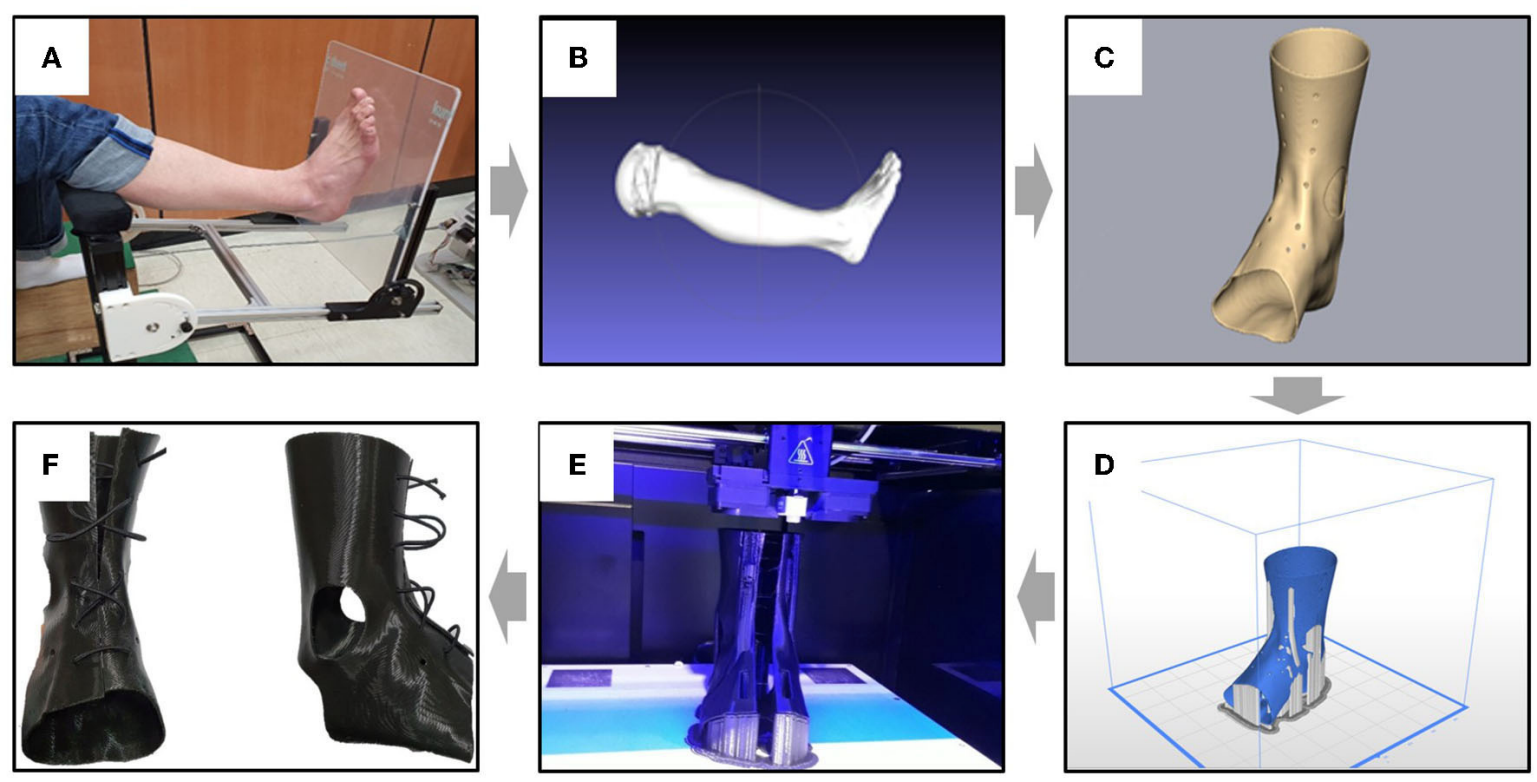
Manufacturing process of customized 3D-printed ankle–foot orthosis (3D-AFO). The process is as follows: **(A)** ankle–foot scanning, **(B)** remeshing from high-resolution image to smooth curved surface, **(C)** individual AFO design with a haptic device, **(D)** 3D printing simulation with print condition setting (infill density: 70%, nozzle thickness: 0.1 mm, layer thickness: 0.19 mm), **(E)** design model output using a 3D printer, and **(F)** final prototype of 3D-AFO.

The initial aim of manufacturing 3D-AFO was to provide a personalized orthosis with improved fit and convenience. The process was divided into three steps (Figure 1): 1) 3D scanning (13): A portable 3D scanner (EinScan Pro 2x, SHINING 3D, San Leandro, United States) captured the hemiparetic areas of the lower limb to generate the initial AFO design. The images were obtained with the patient lying supine, and the target leg was supported by a tripod. The high-resolution image file was remeshing through MeshLab to obtain a smooth curved surface. 2) 3D designing: Unwanted surfaces for 3D printing, such as the medial and lateral malleolus, heel of the foot, and forefoot, were removed from the images using the 3D system's Geomagic Freeform Plus program and phantom haptic device. 3) 3D printing: The designed AFO model was printed in Z-FLEX filament, which is a thermoplastic polyester elastomer with excellent interlayer adhesion and precision of dimensional tolerance, using a 3D printer (Zortrax M300 plus, Olsztyn, Poland). Finally, unnecessary supports of the printed AFO were removed, the center line was cut off, and the length of the band connecting the hole along the center line was adjusted with a boa dial.

### 2.3. Training

**Figure 2:**
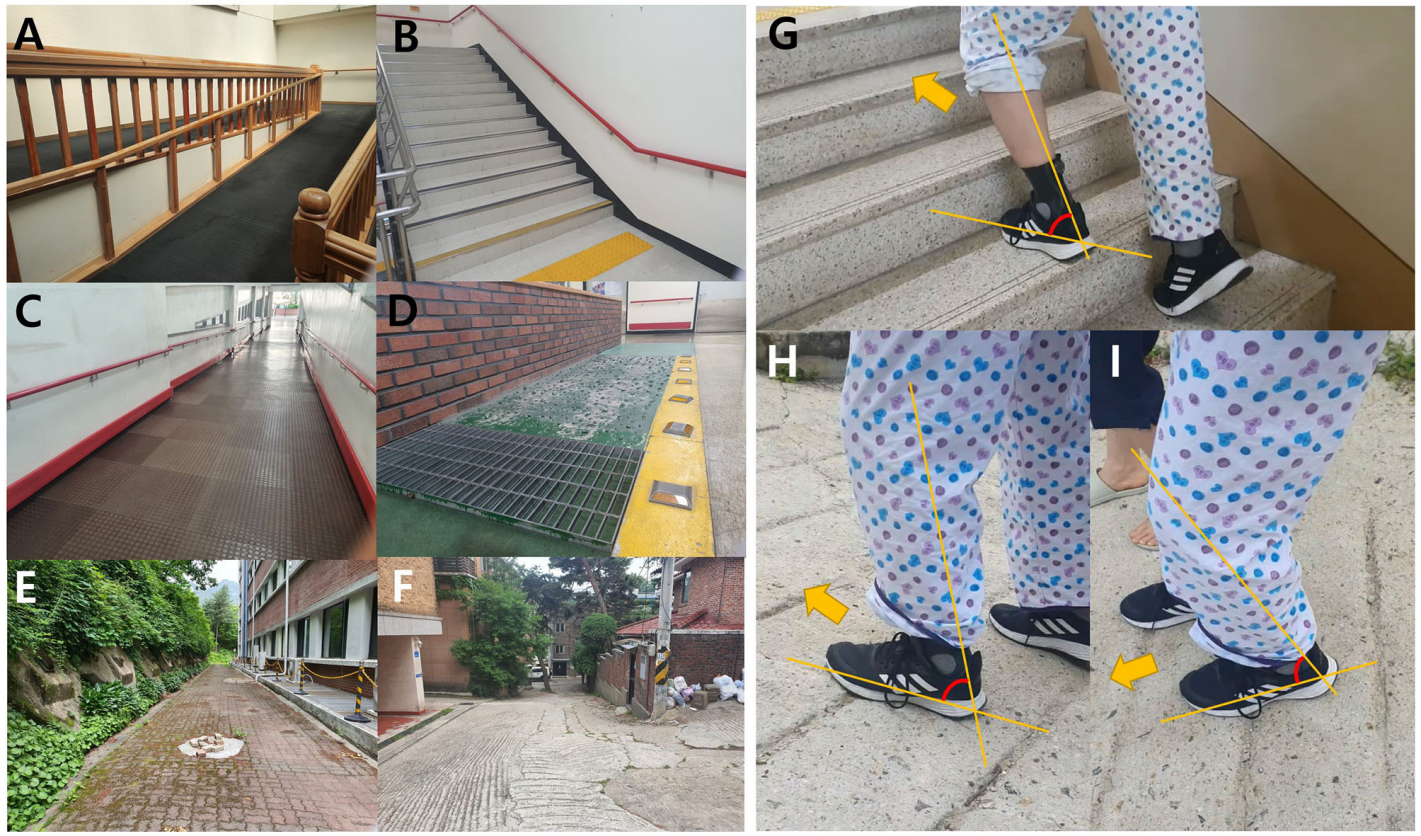
Community gait training consisting of walking on even and uneven terrains, obstacles, slopes, curves, and stairs. The subjects were trained to walk on the ramp **(A, C)** and stairs **(B, G)** for at least 1 week in an indoor environment. After that, for the remaining 3 weeks, the training of walking on uneven surfaces **(D–F)**, climbing the slope **(H)**, and going down the slope **(I)** was gradually increased in the outdoor community environment.

After baseline assessments, the patients performed gait training with 3D-AFO (cases 1 and 2) or conventional AFO (case 3) in various community settings (Figure 2). The intervention program consisted of gait training on even/uneven terrains, curbs, and slopes for 20 min, followed by stair ambulation for 20 min at a progressive walking level per week. The training was performed for 40 min per session for a total of 20 sessions for 4 weeks.

### 2.4. Assessments

The gait was evaluated under three walking (even, uneven, and stair ascent/descent) and four AFO (barefoot, only shoes, shoes with AFOs, and shoes with 3D-AFOs) conditions before and after the intervention (14). To compare differences in gait kinematics according to AFO conditions, all subjects underwent the same gait assessment process with four conditions, i.e., bare foot, shoe, conventional AFO, and 3D-AFO, under three walking conditions at the initial evaluation, i.e., on even and uneven surface and stair. After the sufficient walking adaptation periods, the patients performed a minimum of three trials under each randomized condition, and a minimum of three steps per trial were recorded. The participants did not take the AFOs and shoes off until all gait assessments were completed. For walking on even and uneven surfaces, the patients walked on a 1.5 × 10 m walkway covered with industrial carpeting at a comfortable speed. The uneven surface was created using randomly arranged triangular wooden prisms (H 1.5 cm × W 3.5 cm × L 6–12 cm) placed under a 1.5 × 10 m strip of industrial carpeting with a surface texture identical to that of the even surface. During the stair (17.5 cm riser, 30 cm tread, and 90 cm width) ascent and descent, handrails were present for safety, but they were lightly gripped to prevent weight shift. As footdrop or increased ankle muscle tone could seriously affect the patients' safety, barefoot stair walking was not performed.

To capture kinematic data, 20 reflective markers were placed on each side of the lower limbs. A 12-camera motion capture system (VICON, UK), sampled at 100 Hz, was used. Motion data were low-pass filtered at 6 Hz with a fourth-order Butterworth filter. Surface electromyography (EMG; Delsys Trigno Wireless EMG, Delsys, USA) was performed at 2,000 Hz on each side of the quadriceps (rectus femoris, Q), hamstring (biceps femoris, H), tibialis anterior (TA), and medial gastrocnemius (MG) muscles. The EMG data were processed using a 20–400 Hz band-pass filter and rectification and normalized by maximum voluntary isometric contraction.

Since stroke is the third leading cause of disability in developed countries and the sixth leading cause throughout the world (15), it was one of the first health conditions to receive attention that consider the international classification of functioning, disability, and health (ICF). The ICF model represents a new paradigm with a broader biopsychosocial approach that considers not only the health condition but all factors that can exert a positive or negative influence on functioning (16). Therefore, physical impairments, activity limitations, and social participation according to the ICF model were assessed before and after the training. For the physical impairments, MVIC of paretic ankle muscles was measured using a portable manual muscle strength tester (Lafayette, USA, 2018). The isometric strength of the ankle dorsiflexors, plantar flexors, invertors, and evertors was measured for 5 s, and the maximum value was recorded. For the activity limitations, Fugl–Meyer lower extremity (FM-L), BBS, and a 6-min walking test were performed. The motor domain of FM-L includes measurements of movement, coordination, and reflex action of the hip, knee, and ankle (17). The domain is rated on a 3-point ordinal scale (0 = cannot be performed, 1 = partially performed, and 2 = fully performed). The maximum possible score of the motor domain of FM-L is 34, corresponding to full sensorimotor recovery. BBS was used as a clinical test of a subject's static and dynamic balance (18). The test comprised a set of 14 simple balance-related tasks, ranging from standing up from a sitting position to standing on one foot. The 6-min walking test is commonly used as a measure of walking endurance and a significant predictor of community ambulation and integration in individuals with stroke (19). For social participation, the stroke impact scale, fall efficacy scale, and Beck Depression Inventory were considered. The stroke impact scale participation domain, which includes selected items from the hand function, activity of daily living/instrumental activity of daily living, and mobility domains, can be used as stand-alone scales to assess social and physical function (20). The fall efficacy scale was applied to ascertain a person's level of confidence in performing activities of daily living (21). It is a self-reported questionnaire and contains 10 items, with each scored on a scale of 0–10, and the total summed scores range from 0 to 100. A high score indicates high confidence in performing activities of daily living without falling. The Beck Depression Inventory is a 21-item questionnaire commonly used in research on post-stroke depression (22).

Patient satisfaction with wearing 3D-AFO was investigated using the system usability scale (SUS) and open-ended questions (23).

### 2.5. Data analysis

All the motion and EMG data were exported using Visual 3D software (C-Motion, USA). We analyzed spatiotemporal parameters, joint kinematics, and integrated EMGs and calculated the ankle muscles co-contraction index (CI), which indicates muscle efficiency wherein the antagonist and agonist muscles (i.e., tibialis anterior and medial gastrocnemius) were activated in stance and swing phases (24):


$$CI=\frac{\int_{t1}^{t_{2}}EMG_{TA}\left(t\right)dt}{\int_{t_{1}}^{t_{2}}\left[EMG_{TA}+EMG_{MG}\right]\left(t\right)dt}\times 100.$$

The data were averaged and compared among three walking and four AFO conditions.

## 3. Results

### 3.1. Initial effect

**Figure 3:**
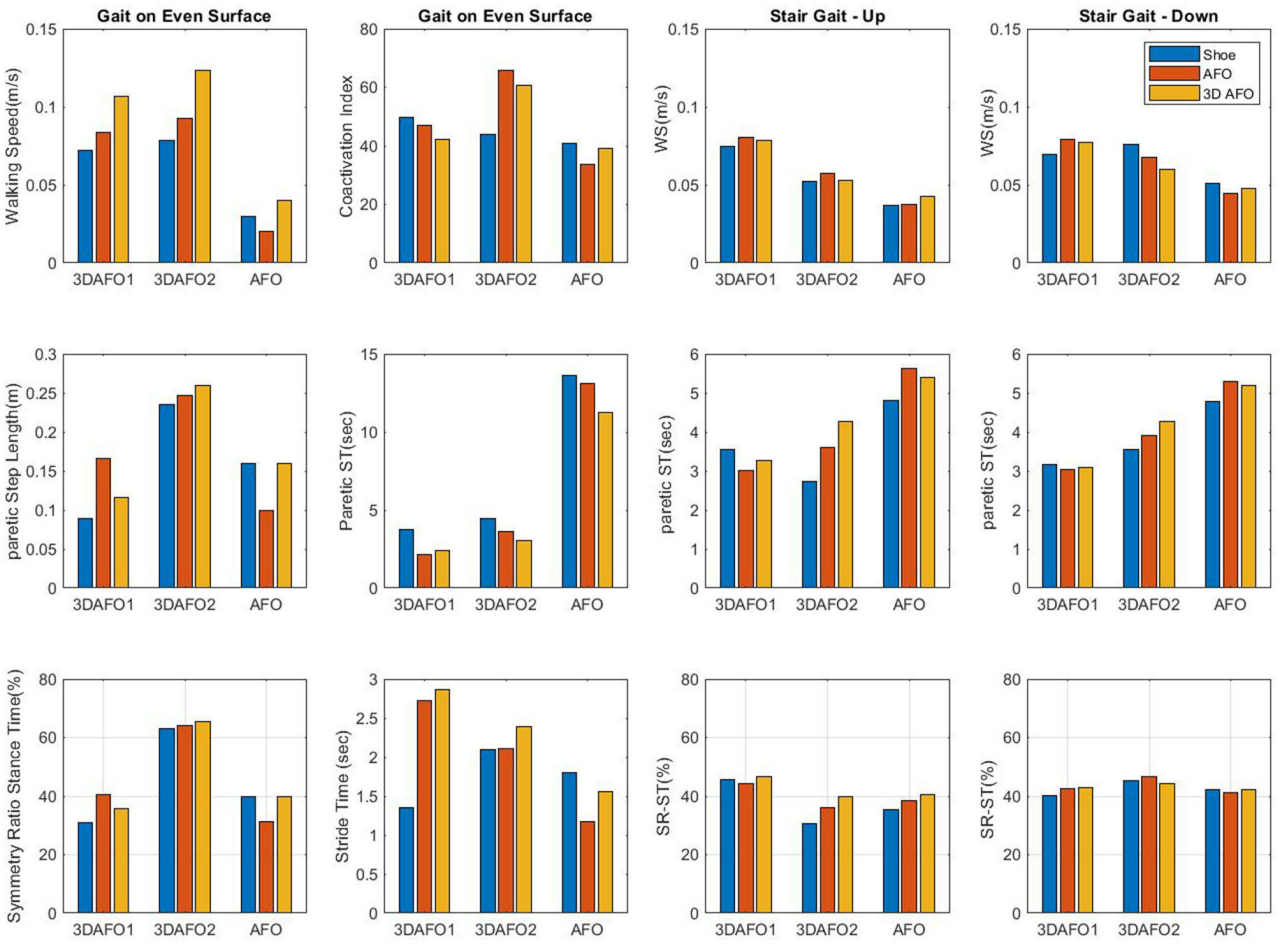
Comparison of spatiotemporal gait kinematics in different AFO (ankle–foot orthosis) conditions. AFO, ankle–foot orthosis; 3D-AFO, three-dimensional-printed ankle–foot orthosis; CI, co-contraction index; Q, quadriceps; H, hamstrings; TA, tibialis anterior; GCM, gastrocnemius.

As a result of initial gait assessments of the three participants, the walking speed and step length increased more in 3D-AFO conditions, followed by AFO and only shoes (Figure 3 and Supplementary Table 2) conditions. Stride width increased more in the 3D-AFO condition than in the AFO condition. Among all the AFO conditions, 3D-AFO showed the most symmetrical gait. The ankle and thigh muscle CI increased more in the only shoes and AFO conditions than in the 3D-AFO conditions. The ankle ROM increased the most in the only shoes condition, followed by the 3D-AFO and AFO conditions; the increase was more on the even surface than on the uneven surface (Supplementary Figures 1, 2). The knee and hip ROM showed the most increase in the AFO condition. During stair ascents, walking speed increased in the AFO and 3D-AFO conditions compared to that in the only shoes condition. 3D-AFO showed the most symmetrical stance time among other conditions. During stair descents, the only shoes condition showed the most increased walking speed and symmetrical cycle time (Supplementary Table 3).

### 3.2. Long-term effects

**Figure 4:**
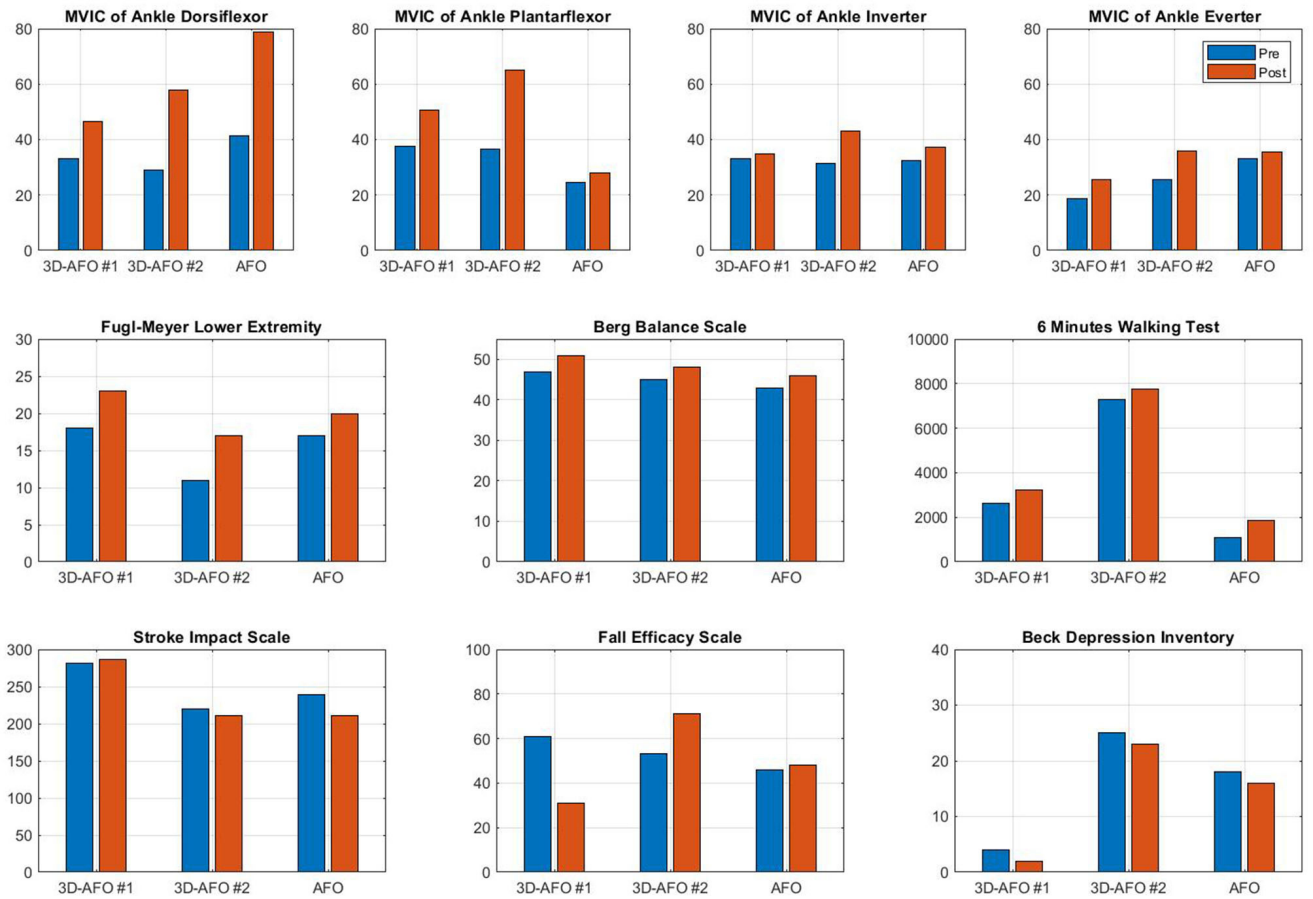
Effects of 4 weeks of gait training with AFO on maximum voluntary isometric contraction force of ankle muscles, Fugl–Meyer assessment of lower extremity, the Berg balance scale, the 6-min walking test, the stroke impact scale, the fall efficacy scale, and the Beck Depression Inventory. Pre, pre-test; Post, post-test; FU, follow-up.

Patients trained with 3D-AFO acquired increased walking speed (difference value; case 1: 0.1 m/s, case 2: 0.04 m/s) and step length (case 1: 0.2 m, case 2: 0.15 m). However, there was no difference in the stride width with an improvement in the symmetry of the cycle and stance time and decreased symmetry of the step length. Patients trained with AFO showed no difference in walking speed, decreased step length (case 3: −0.01 m), increased stride width (case 3: 0.02 m), and decreased gait symmetry. All patients experienced improvements in their physical impairments (elicited by the strength of ankle dorsiflexor) and activity limitations (elicited by the Fugl–Meyer assessment of lower extremity, Berg balance scale, and 6-min walking test); however, social participation did not increase (elicited by the stroke impact scale and fall efficacy scale). All participants showed decreased Beck Depression Inventory scores after 4 weeks (Figure 4).

### 3.3. Patient satisfaction

The average SUS score was 69 (SD: 5.2), and 60% of the respondents reported a score of 71 or higher. A SUS score of >70 indicates good products (23). In the interview using open-ended questions, the respondents were satisfied with the use of 3D-AFO in terms of its weight, thinness, better fit, enhanced aesthetics, safety (stability), and improved functions. Furthermore, they revealed that 3D-AFO was more convenient on uneven surfaces, ramps, and stairs when compared with conventional AFO or only shoes. However, they felt uncomfortable due to the difficulty of wearing the 3D-AFO by themselves.

## 4. Discussion

We investigated the effects of 3D-AFO use on gait kinematics and physical functions of patients with stroke. All the participants had increased step length, stride width, and symmetry; the muscle efficiencies of Q/H and TA/MG were improved during the stance and swing phases, respectively. In particular, stair ambulation with 3D-AFO allowed increased ankle ROM and symmetrical gait when compared with AFO use; this improvement was more effective during the 4-week community ambulation training. This study is unique in that it compared the effects of 3D-AFO, including muscle efficiency and individual satisfaction, in community ambulation.

3D printing technology is advantageous as it maximizes the design freedom to optimize the stiffness properties of AFO (7, 8, 25). The greater AFO stiffness generally results in reduced peak joint angle of ankle plantar flexion and dorsiflexion and increased dorsiflexion at initial contact and total ROM; (5, 26) it also increases the peak ankle dorsiflexion moment and decreases peak knee extension (6). A study that compared different 3D-AFO stiffnesses using various materials reported no significant differences in temporal-spatial parameters and ankle angles, but a difference was noted in the ankle ROM over the whole gait cycle (27). This study also showed increased ankle ROM during stair ascent as well as gait on an even surface. Another study reported that the selective adjusting orthotic stiffness of 3D-AFO can produce stance phase stability, mitigation of toe drag, reduction in steppage gait, and an improvement in symmetry and muscle efficiency (28). In our patients, the step length, stride width, symmetry, and muscle efficiency increased more with 3D-AFO than with conventional AFO, which indicates that a decrease in AFO stiffness helps the user to improve the biomechanical gait function. However, in patients with excessive ankle spasticity that could seriously interfere with gait, walking with AFO was more effective than walking with 3D-AFO. The use of stiff AFO is often accompanied by reduced activity in the paretic ankle muscles and disuse atrophy, causing long-term dependence (29). A previous study compared the muscle activity under different AFO conditions and demonstrated a decrease in CI, which indicates increased muscle efficiency when using dynamic AFO compared with solid AFO (30). Similarly, the stance CI of Q/H and swing CI of TA/MG in 3D-AFO conditions in this study were decreased compared to those of the only shoes and conventional AFO conditions. The TA muscle activity did not differ significantly between the AFO and no orthosis conditions as shown in a previous study (31). Although a few studies have reported muscle efficiency in different AFO stiffnesses, the current study suggests that variations in 3D-AFO stiffness can affect the wearer's biomechanical function and muscle efficiency.

Community environments require adaptations of the lower limb to successfully navigate rough terrain or to ascend and descend slopes and stairs (32). In particular, stair ambulation, which requires higher muscular strength, coordination, and balance (33, 34), is the best predictor of physical activity levels in community-dwelling people with stroke; (33) it requires greater ROM and ankle joint power than level ground walking (35). Although AFO use has the advantage of preventing trips and falls resulting from foot drop and controlling the ground reaction force to reduce fatigue during stair ambulation (2), it commonly increases the risk of fall because of the limited ankle ROM that does not allow control of the upper part of the lower limb both in stair ascent and decent. Nevertheless, prescribing AFO is recommended for the majority of stroke patients. The meta-analysis of 434 stroke patients reported immediate or short-term effectiveness on walking speed, cadence, step length, stride length, timed up and go test, functional ambulation category score, sagittal plane angle at initial contact, and knee sagittal angle at toe-off (*p* < 0.05) (36). However, this study, differing from previous studies, was mainly focused on the effects of AFO in a simulated community environment such as uneven walkway and stair gait in comparison with its long-term training effect. In the present study, the ankle ROM was more limited in both the AFO conditions than in the only shoes condition; it was limited more with conventional AFO than with 3D-AFO during stair ambulation. During stair ascent, 3D-AFO would have helped to generate an appropriate ankle ROM, allowing the tibia to progress over the foot; (37) active ankle plantar flexion and increased power generation during trailing limb push-up (38) resulted in increased ankle torque and knee extensor moment (39). Conversely, the only shoes condition showed a tendency to decrease the stance time, swing time, and cycle time and increase the symmetry of cycle time during stair descent. Controlled ankle dorsiflexion and power absorption, which are critical for weight acceptance during stair descent, are more feasible in the only shoes condition (37). Nevertheless, 3D-AFO indicated the most symmetric stance time among the other three conditions during stair ascents and more symmetric stance time when compared with AFO condition during stair descent. This indicates that 3D-AFO may be more useful in community environments including uneven terrain, stairways, and slopes; therefore, it is closely linked to social participation.

Satisfaction with AFO wear often has a significant impact on the user's physical function. The users require improved size, weight, adjustability, and durability as well as overall biomechanical function of their AFOs (3). Although the safety and effectiveness of AFO are considered the most important, some individuals, especially adolescents, prioritize the aesthetic and psychological factors (2, 3). A participant of this study, who was planning to return to school as a teacher, was obsessed with the symmetrical gait pattern without an outstanding conventional AFO wear. Another participant complained that conventional AFO was bulky, requiring different sizes for both shoes, which led to the development of pain, blisters, and calluses. The participants were satisfied with 3D-AFO's thinness, light weight, and comfortable feeling with wearing shoes. Conversely, they also gave feedback on decreased durability of 3D-AFO when used continuously for more than 2 months; patients with hemiparesis found difficulty in wearing it alone. Nevertheless, all the participants were satisfied with the usability of 3D-AFO when they returned to the community and their colleagues. However, social participation did not improve among our study participants. The flexibility of 3D printing material, i.e., thermoplastic polyurethane, allowed more range of motion of the ankle joint in 3D-AFO, which enabled more comfortable walking in a community environment where ramps and stair climbing were often unavoidable. In addition, this study deduced that the function of AFO in the community is not only in the enhancement of biomechanical function but also in the aesthetic and psychological factors, and better fit would be a critical factor to stroke survivors.

This study has some limitations. First, there may be baseline functional differences between the participants that might have affected the outcomes. Second, the number of participants wearing 3D-AFO or AFO during the 4-week training and their initial gait functions were different. Therefore, the intensity of community ambulation training such as the number of stairs going up and down and walking distance were not equal for all patients. Third, actual AFO stiffness was not measured. Fourth, the effect of wearing 3D-AFO in this study was applied to only three stroke patients, so it cannot be generalized to all stroke patients. Further studies are required to investigate the effect of 3D-AFO and its stiffness on a larger number of patients with chronic stroke while considering the age, gender, footwear, and lifestyle of the users.

## 5. Conclusion

The participants were satisfied with 3D-AFO use. 3D-AFO was particularly effective in increasing the step length, stride width, symmetry, ankle ROM, and muscle efficiency during gait on even surfaces and stair ascent. After the 4-week training, the patients who trained with 3D-AFO showed an increase in ankle strength, balance, gait endurance, and gait symmetry and a reduction in depression; however, their social participation did not improve.

## 6. Patient perspectives

Case 1: The patient and his caregiver revealed that the 3D-AFO was especially helpful while walking on ramps and climbing stairs. However, when he wanted to walk faster on the treadmill, the AFO could hold the paretic ankle more firmly than the 3D-AFO. He was very satisfied with the 3D-AFO and reported its continuous use after returning home.

Case 2: At the end of the training, his improved gait function stood out conspicuously, but his gait speed slowed slightly because he was paying attention to an excessively symmetrical gait pattern. He was satisfied not only with the effectiveness but also with the aesthetics of the 3D-AFO and the ease of wearing it with shoes.

Case 3: The patient's ankle spasticity worsened especially while standing and walking. He liked the better fit and flexibility of the 3D-AFO but preferred to wear conventional AFO, which is more stable and robust.

## Data availability statement

The original contributions presented in the study are included in the article/Supplementary material, further inquiries can be directed to the corresponding author.

## Ethics statement

The studies involving human participants were reviewed and approved by Institutional Review Board at National Rehabilitation Center on February 2nd, 2021 (IRB No. NRC-2021-01-002). The patients/participants provided their written informed consent to participate in this study. Written informed consent was obtained from the individual(s) for the publication of any potentially identifiable images or data included in this article.

## Author contributions

J-EC, K-JS, and HK contributed to the study design. J-EC and K-JS contributed to the project administration. SH acquired the data. J-EC and SH performed the data analysis. J-EC contributed to writing—original draft preparation and implementation of evaluations and interventions. K-JS contributed to the fabrication of 3D-AFO and the implementation of evaluations. K-JS, SH, and HK were involved in writing, reviewing, and editing. HK contributed to funding acquisition and contributed to the conception and methodology. All authors contributed to the article and approved the submitted version.
