# Supplementary material for: Effects of community ambulation training with 3D-printed ankle–foot orthosis on gait and functional improvements: a case series of three stroke survivors
Source: Front Neurol. 2023 May 31;14:1138807. doi: 10.3389/fneur.2023.1138807 (PMC10264639; doi:10.3389/fneur.2023.1138807)

Supplementary Material

Case reports: Effect of community ambulation with 3D-printed ankle-foot-orthoses on gait and social participation of patients with stroke

Ji-Eun Cho^1^†, Kyeong-Jun Seo^1^, Sunghe Ha^2^, Hogene Kim^3*^

*** Correspondence:** Hogene Kim: [hogenekim@gmail.com](mailto:hogenekim@gmail.com)

# Supplementary Tables

## Supplementary Table 1. Baseline characteristics of the study participants

## Supplementary Table 2. Comparison of gait parameters under four ankle-foot orthosis conditions

## Supplementary Table 3. Comparison of kinematics during stair ascent and descent under four ankle-foot orthosis conditions.

# Supplementary Figures

## Supplementary Figure 1. Joint angles of subjects with chronic stroke during even walking and stair ambulation under AFO conditions

## Supplementary Figure 2. Ankle, knee, and hip angles in patients with chronic stroke during walking on even and uneven surfaces under three different AFO conditions

**
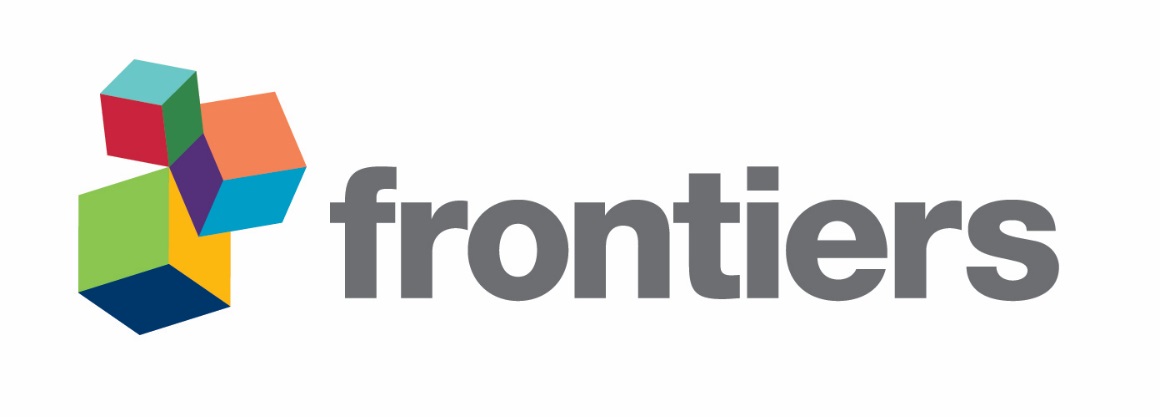
**

**1.1 Supplementary Table 1.** Baseline characteristics of the study participants

| Characteristics | | Case 1 | Case 2 | Case 3 |
| --- | --- | --- | --- | --- |
| Age (y) | | 30 | 58 | 47 |
| Sex | | Male | Male | Male |
| Weight (kg) | | 60 | 65 | 80 |
| Height (cm) | | 172 | 168 | 174 |
| Time post stroke (months) | | 10.5 | 24.4 | 21.2 |
| Affected side | | Left | Left | Left |
| Diagnosis | | Rt. Basal ganglia ICH | Multifocal scattered infarction | Rt. Putamen ICH & IVH |
| Modified Ashworth Scale of ankle joint muscles (0/1/1+/2) | | 1 | 1 | 1+ |
| MMSE (score) | | 29 | 27 | 25 |
| ROM of the ankle (°) | DF | 20.5 | 22.3 | 20.0 |
|  | PF | 46.0 | 39.5 | 37.0 |
|  | INV | 40.7 | 44.6 | 41.0 |
|  | EV | 39.2 | 37.1 | 38.9 |
| Manual muscle test (score) | DF | 2.5/5 | 1/5 | 2/5 |
|  | PF | 1.5/5 | 1.5/5 | 1.5/5 |
|  | INV | 2/5 | 1/5 | 1.5/5 |
|  | EV | 2.5/5 | 1/5 | 1.5/5 |
| Strength of the ankle (N) | DF | 46.5 | 29.0 | 41.4 |
|  | PF | 50.8 | 36.5 | 24.7 |
|  | INV | 35.0 | 31.5 | 32.6 |
|  | EV | 25.6 | 25.6 | 33.2 |
| Fugl-Meyer scale (score) | | 18 | 11 | 17 |
| Functional ambulation category (score) | | 3 | 4 | 3 |
| Berg balance scale (score) | | 47 | 45 | 43 |
| Timed up & go (sec) | | 52.4 | 61.5 | 340.4 |
| Walking speed (cm/sec) | | 7.2 | 7.8 | 3.0 |
| Fall efficacy scale (score) | | 61 | 53 | 46 |
| Stroke Impact Scale (score) | | 281 | 220 | 239 |
| Back Depression Inventory (score) | | 4 | 25 | 18 |

Abbreviations: DF, dorsiflexion; EV, eversion; ICH, intracerebral haemorrhage; INV, inversion; IVH, intraventricular haemorrhage; MMSE, Mini-Mental State Examination; PF, plantarflexion; Rt, right; ROM, range of motion.

**1.2 Supplementary Table 2.** Comparison of gait parameters under four ankle-foot orthosis conditions

|  |  | Spatiotemporal parameters | | | | | | | Muscle efficiency | | | | |
| --- | --- | --- | --- | --- | --- | --- | --- | --- | --- | --- | --- | --- | --- |
| AFO Conditions | Participants | Walking speed (m/sec) | Step length (cm) | Stride width (cm) | Cycle time (sec) | Stance time (sec) | Swing time (sec) | Symmetry (step length, %) | | Stance CI (T/G) | Stance CI (Q/H) | Swing CI (T/G) | Swing CI (Q/H) |
| Bare foot | Case 1 | 0.07 | 10.7 | 20.7 | 3.7 | 3.3 | 0.5 | 45.0 | | 43.5 | 50.1 | 57.3 | 35.0 |
|  | Case 2 | 0.17 | 26.0 | 19.4 | 2.7 | 2.1 | 0.5 | 65.9 | | 52.3 | 50.7 | 81.9 | 41.0 |
|  | Case 3 | 0.02 | 3.0 | 13.0 | 19.2 | 18.7 | 0.5 | 11.54 | | 28.6 | 39.6 | 61.9 | 39.7 |
|  | Total | 0.08 (0.07) | 13.2 (11.7) | 17.7 (4.1) | 8.6 (9.3) | 8.0 (9.3) | 0.5 (0.0) | 40.8 (27.4) | | 41.5 (11.9) | 46.8 (6.3) | 67.0 (13.1) | 38.5 (3.1) |
| Only shoes  AFO | Case 1 | 0.07 | 8.9 | 23.7 | 4.4 | 3.7 | 0.6 | 31.0 | | 49.8 | 48.6 | 71.3 | 37.8 |
|  | Case 2 | 0.08 | 23.5 | 18.2 | 5.1 | 4.5 | 0.6 | 63.1 | | 44.0 | 64.9 | 72.9 | 62.4 |
|  | Case 3 | 0.03 | 16.0 | 19.0 | 14.5 | 13.6 | 0.8 | 40.0 | | 40.8 | 36.8 | 73.4 | 27.5 |
|  | Total | 0.06 (0.03) | 16.1 (7.3) | 20.3 (3.0) | 8.0 (5.6) | 7.3 (5.5) | 0.7 (0.1) | 44.7 (16.6) | | 44.9 (4.6) | 50.1 (14.1) | 72.5 (1.1) | 42.5 (17.9) |
| AFO  3D-AFO | Case 1 | 0.15 | 16.6 | 20.7 | 2.8 | 2.2 | 0.6 | 40.4 | | 47.1 | 43.5 | 65.2 | 40.0 |
|  | Case 2 | 0.09 | 24.7 | 17.7 | 4.3 | 3.6 | 0.7 | 64.0 | | 65.7 | 40.0 | 79.2 | 41.9 |
|  | Case 3 | 0.02 | 10.0 | 14.0 | 13.9 | 13.1 | 0.8 | 31.3 | | 33.5 | 47.3 | 69.9 | 24.9 |
|  | Total | 0.09 (0.07) | 17.1 (7.4) | 17.5 (3.3) | 7.0 (6.0) | 6.3 (5.9) | 0.7 (0.1) | 45.2 (16.9) | | 48.8 (16.1) | 43.6 (3.6) | 71.4 (7.1) | 35.6 (9.3) |
| 3D-AFO | Case 1 | 0.11 | 11.6 | 23.4 | 2.9 | 2.4 | 0.6 | 35.9 | | 42.2 | 42.8 | 63.6 | 34.8 |
|  | Case 2 | 0.12 | 25.9 | 18.7 | 3.6 | 3.0 | 0.6 | 65.5 | | 60.8 | 46.3 | 77.6 | 41.6 |
|  | Case 3 | 0.04 | 16.0 | 17.0 | 11.9 | 11.2 | 0.7 | 40.0 | | 39.2 | 35.6 | 68.1 | 34.6 |
|  | Total | 0.09 (0.04) | 17.8 (7.3) | 19.7 (3.3) | 6.2 (5.0) | 5.5 (4.9) | 0.6 (0.1) | 47.1 (16.0) | | 47.4 (11.7) | 41.6 (5.5) | 69.8 (7.2) | 37.0 (4.0) |

Total values are written as mean (standard deviation).

Abbreviations: AFO, ankle-foot orthosis; 3D-AFO, three-dimensional-printed AFO; CI, co-contraction index; Q, quadriceps; H, hamstrings; T, tibialis anterior; G, gastrocnemius.

**1.3 Supplementary Table 3.** Comparison of kinematics during stair ascent and descent under four ankle-foot orthosis conditions.

|  |  | Stair ascent | | | | | | Stair descent | | | | | | |  |
| --- | --- | --- | --- | --- | --- | --- | --- | --- | --- | --- | --- | --- | --- | --- | --- |
| AFO Conditions | Participants | Walking speed (m/sec) | Cycle Time (sec) | Stance Time (sec) | Swing Time (sec) | Symmetry | | | Walking speed (m/sec) | Cycle Time (sec) | Stance Time (sec) | Swing Time (sec) | Symmetry | | |
|  |  |  |  |  |  | Cycle Time (%) | Stance Time (%) | |  |  |  |  | Cycle Time (%) | Stance Time (%) | |
| Only shoes | Case 1 | 0.07 | 4.9 | 3.5 | 1.4 | 50.7 | 45.7 | | 0.07 | 5.2 | 3.2 | 2.0 | 49.9 | 40.2 | |
|  | Case 2 | 0.05 | 6.6 | 2.7 | 3.9 | 49.4 | 30.6 | | 0.08 | 4.8 | 3.6 | 1.3 | 50.7 | 45.5 | |
|  | Case 3 | 0.04 | 9.0 | 4.8 | 4.2 | 48.7 | 35.4 | | 0.05 | 7.1 | 4.8 | 2.3 | 49.9 | 42.2 | |
|  | Total | 0.05 (0.02) | 6.9 (2.1) | 3.7 (1.0) | 3.2 (1.6) | 49.6 (1.0) | 37.2 (7.7) | | 0.07 (0.01) | 5.7 (1.2) | 3.8 (0.8) | 1.9 (0.5) | 50.2 (0.5) | 42.6 (2.7) | |
| AFO | Case 1 | 0.08 | 4.6 | 3.0 | 1.6 | 51.2 | 44.1 | | 0.08 | 4.8 | 3.0 | 1.8 | 50.3 | 42.4 | |
|  | Case 2 | 0.06 | 5.5 | 3.6 | 1.9 | 44.3 | 36.1 | | 0.07 | 5.5 | 3.9 | 1.6 | 52.4 | 46.6 | |
|  | Case 3 | 0.04 | 8.8 | 5.6 | 3.2 | 47.6 | 38.6 | | 0.04 | 8.4 | 5.3 | 3.1 | 50.9 | 41.2 | |
|  | Total | 0.06 (0.02) | 6.3 (2.2) | 4.1 (1.4) | 2.2 (0.8) | 47.7 (3.5) | 39.6 (4.1) | | 0.06 (0.02) | 6.2 (1.9) | 4.1 (1.1) | 2.2 (0.8) | 51.2 (1.1) | 43.4 (2.8) | |
| 3D-AFO | Case 1 | 0.08 | 4.8 | 3.3 | 1.6 | 52.4 | 46.6 | | 0.08 | 4.9 | 3.1 | 1.8 | 51.7 | 42.9 | |
|  | Case 2 | 0.05 | 6.2 | 4.3 | 1.9 | 46.6 | 39.8 | | 0.06 | 6.2 | 4.3 | 1.9 | 51.1 | 44.2 | |
|  | Case 3 | 0.04 | 8.2 | 5.4 | 2.8 | 48.8 | 40.6 | | 0.05 | 7.4 | 5.2 | 2.2 | 49.2 | 42.3 | |
|  | Total | 0.06 (0.02) | 6.4 (1.7) | 4.3 (1.1) | 2.1 (0.6) | 49.3 (2.9) | 42.3 (3.7) | | 0.06 (0.01) | 6.2 (1.2) | 4.2 (1.1) | 2.0 (0.2) | 50.7 (1.3) | 43.1 (1.0) | |

Total values are written as mean (standard deviation).

Abbreviations: AFO, ankle-foot orthosis; 3D-AFO, 3D-printed AFO.

**2.1 Supplementary Figure 1.**

When patients with chronic stroke walked with shoes or 3D-printed ankle-foot orthosis (3D-AFO), the range of motion of the ankle joint appeared to increase more than when they walked with ankle-foot orthosis (AFO). During stair climbing, the ankle angle showed greater plantarflexed in the following order: 3D-AFO and AFO. The knee joint was more flexed during walking with AFO on an even surface. However, the knee joint was most flexed when they wore shoes while climbing stairs.


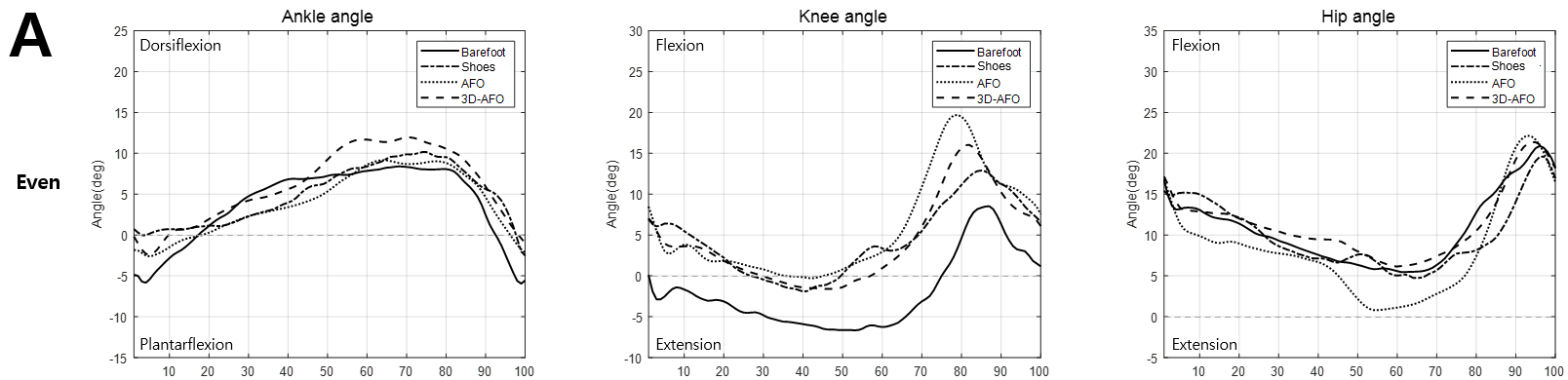


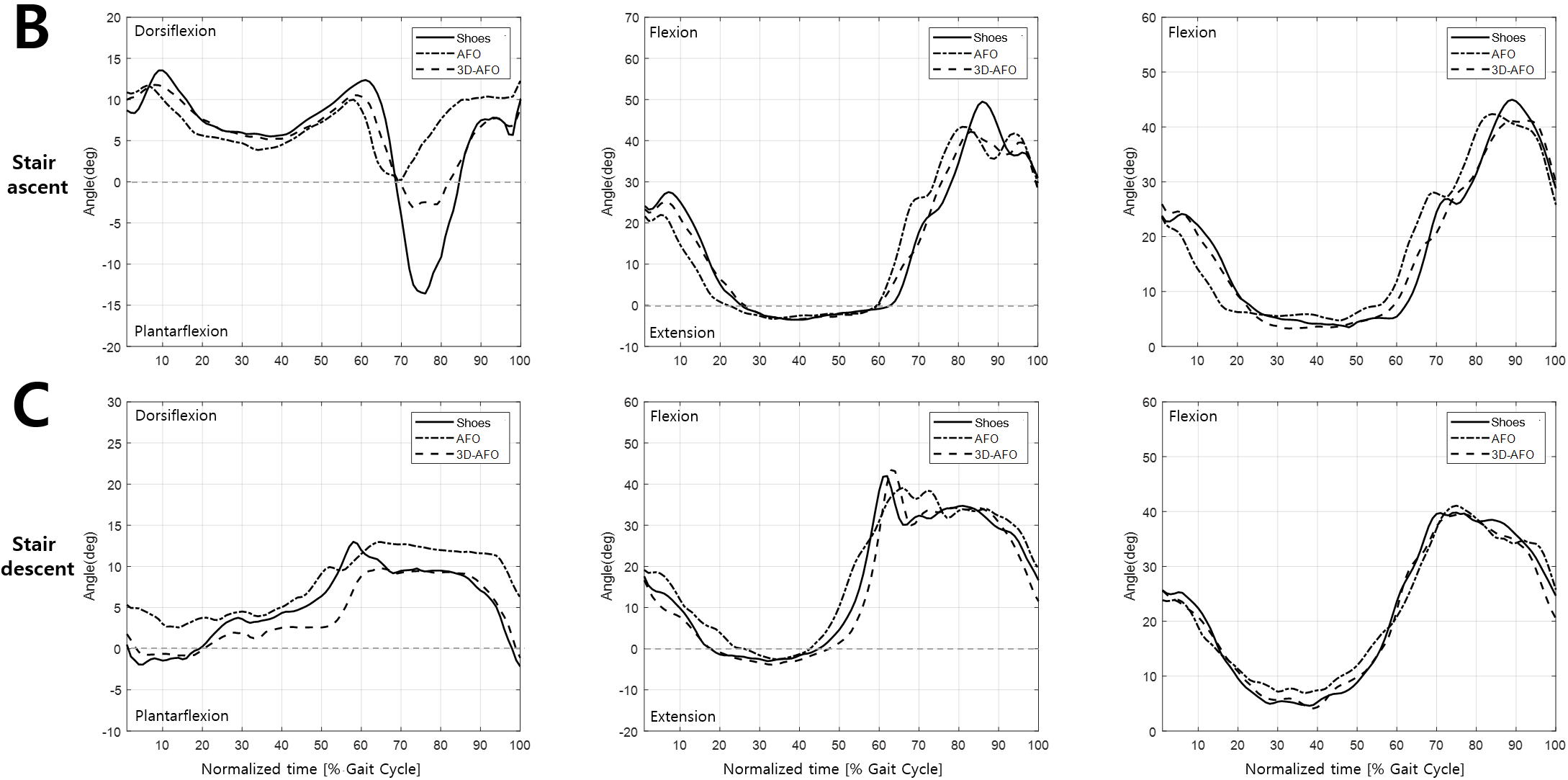


**2.2 Supplementary Figure 2.**

When patients walked on an even or uneven surface under different AFO conditions, the ankle angle during even walking was greater than that during uneven walking only in footwear and 3D-AFO conditions. While patients walked with AFO, ankle angles were similar in even and uneven walking, and knee angles appeared to be more flexed with AFO compared to other conditions.


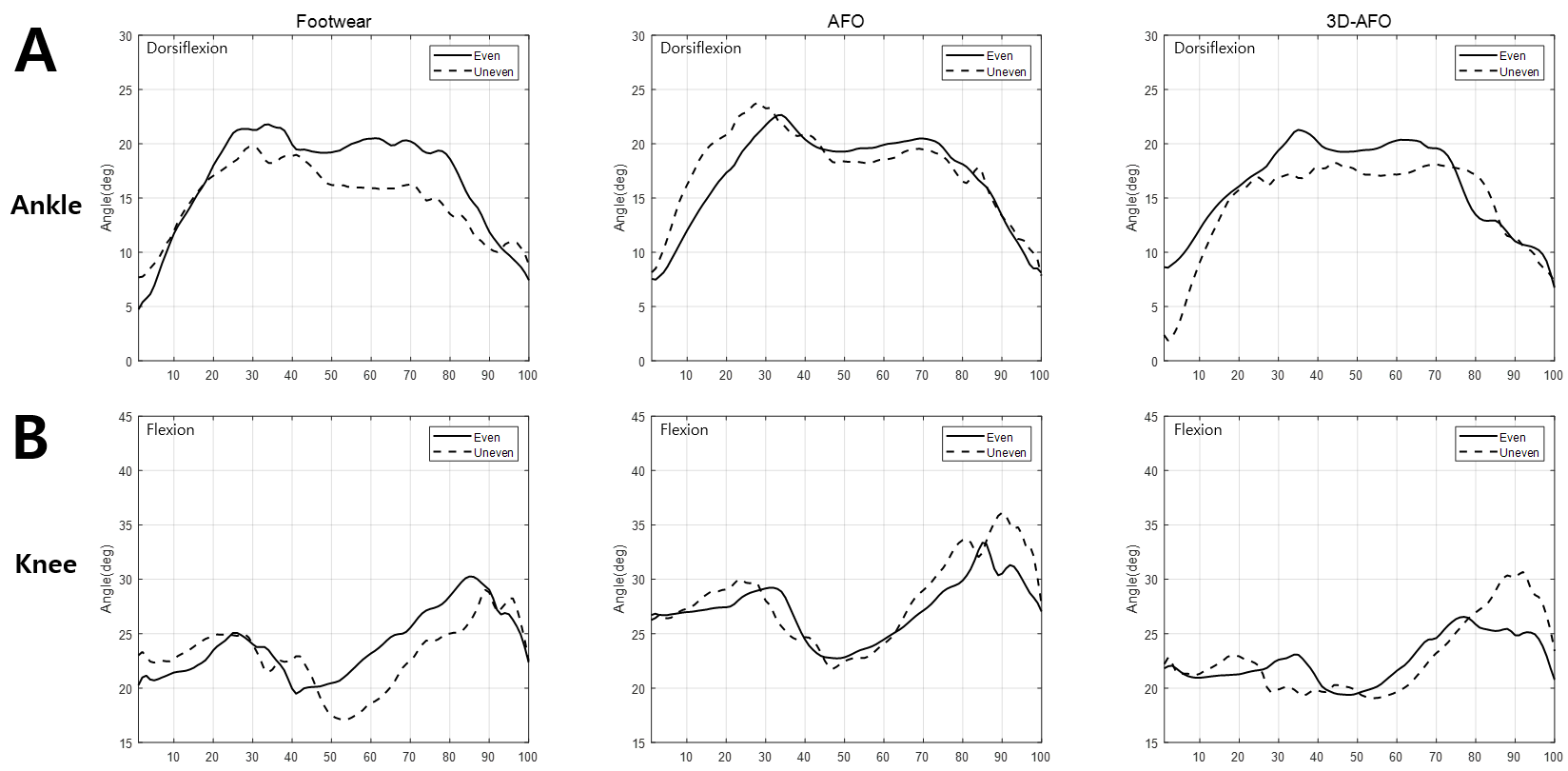


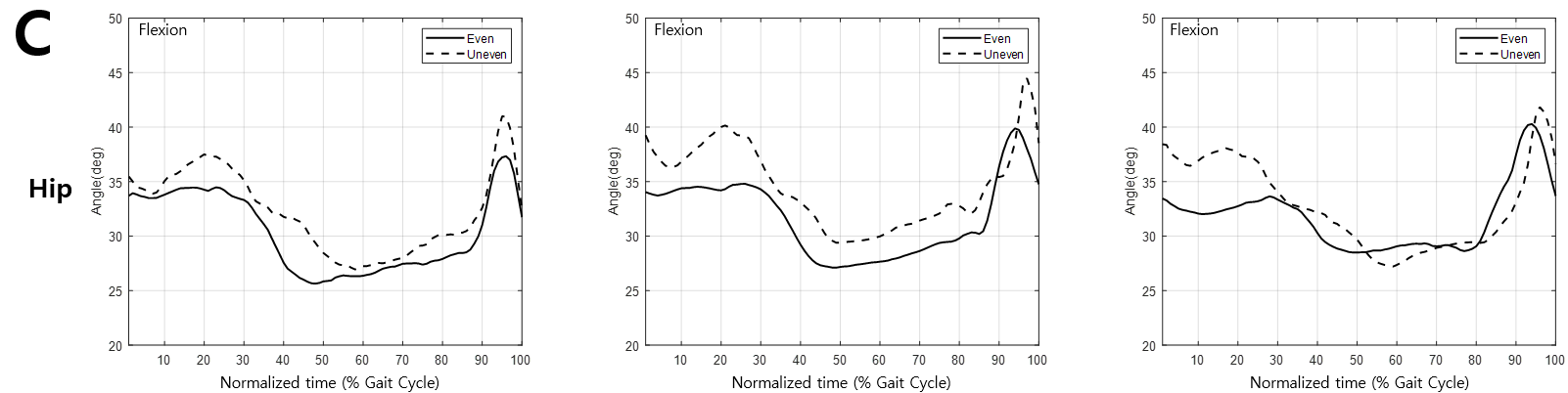

Supplement: Supplementary file 1 [file Data_Sheet_1.docx]
